# Near-field projection optical microscope (NPOM) as a new approach to nanoscale super-resolved imaging

**DOI:** 10.1038/s41598-023-41978-6

**Published:** 2023-09-16

**Authors:** Abhijit Sanjeev, David Glukhov, Rinsa Salahudeen Rafeeka, Avi Karsenty, Zeev Zalevsky

**Affiliations:** 1 Faculty of Engineering, Bar-Ilan University, 5290002 Ramat Gan, Israel; 2 Nanotechnology Center, Bar-Ilan University, 5290002 Ramat Gan, Israel; 3 Lev Academic Center, Faculty of Engineering, Advanced Lab. of Electro-Optics (ALEO), 9116001 Jerusalem, Israel; 4 Nanotechnology Educational and Research Center, Lev Academic Center, 9116001 Jerusalem, Israel

**Keywords:** Applied optics, Optical techniques

## Abstract

A new super-resolution method, entitled Near-field Projection Optical Microscopy (NPOM), is presented. This novel technique enables the imaging of nanoscale objects without the need for surface scanning, as is usually required in existing methods such as NSOM (near-field scanning optical microscope). The main advantage of the proposed concept, besides the elimination of the need for a mechanical scanning mechanism, is that the full field of regard/view is imaged simultaneously and not point-by-point as in scanning-based techniques. Furthermore, by using compressed sensing, the number of projected patterns needed to decompose the spatial information of the inspected object can be made smaller than the obtainable points of spatial resolution. In addition to the development of mathematical formalism, this paper presents the results of a series of complementary numerical tests, using various objects and patterns, that were performed to verify the accuracy of the reconstruction capabilities. We have also performed a proof of concept experiment to support the numerical formalism.

## Introduction

Near-field sensing and super-resolution became the hot domains of research in nanotechnology research and activities. In particular, surface analysis became a challenging domain of investigation, mainly due to increasingly complex needs. Biological and chemical sensing look to analyze substances and residues. A large variety of application domains exist, starting from material science, pharmaceutical, drug, and explosives detection and until COVID-19 variant presence detection. Indeed, atomic and molecular level of material properties determined with specific surface approaches requires today's experimental techniques combined with computational methods. The decoding of material residues on a surface can serve sometimes as the decoding key for an event or a contamination propagation. Forensic science is one of the best customers of such analyzing methods. Of course, non-destructive evaluations (NDE) are preferable to keep the original sample.

In the field of super-resolution, important contributions and inventions were published in the last two decades^1,2^. More specifically, special methods were invented like structured illumination^3–5^, localized microscopy super-resolved imaging ^6–8^, non-linear microscopy^9^, and the single pixel-based imaging^10^.

In the domain of near-field, several families of techniques do exist and are used according to the needs. The two famous core methods of surface scanning – AFM and NSOM – were developed separately in the 1980s, forty years later the trend is clearly to improve, combine, and adapt methods while reducing efforts and increasing accuracy. Invented in 1986 by IBM scientists^11^, and following its Scanning Tunneling Microscope (STM) predecessor^12,13^, the Atomic Force Microscopy (AFM) served for years as the Scanning Probe Microscopy (SPM) branch ^14,15^ core method^16^, mainly used for nanoscale surface topography characterization. On the other hand, the Near Field Scanning Optical Microscopy (NSOM)^17^, served in parallel as the sub-diffractive optical characterization core method^18^. With time, enhanced variations of these techniques were developed^19,20^.

Atomic Force Microscope (AFM) enables to map of the topography of a surface, the analysis of topographical parameters^21^, and the profile of nano-particles with cross-sectional views^22^, while scanning it with a nanoscale tip. The topography is possible in nano-metric resolution due to the tip itself: When it stands in a nano-metric distance from the surface, several forces are interacting on it, among others Van der Waals forces^23^, Casimir forces^24,25^, capillary forces^26^, and electrostatic forces^27^. Due to these interactions between the scanned surface and the tip, when the tip approaches the surface, it is moved aside due to these forces. From the vibrations/moves of the tip, it is possible to map the scanned sample topography. The tip’s vibrations are measured by a laser beam, which illuminates it. When the reflections change because of the vibrations, then the reading of the equipment changes accordingly.

When compared to AFM, Near-Field Scanning Optical Microscope (NSOM) provides an optical image (i.e. not a topographical/mechanical image) of the surface^28^. Its tip extremity, which is significantly smaller than the optical wavelength, records evanescent waves reflected (or transmitted) from the surface^29,30^ by tiny spatial structures, which are smaller than the optical wavelength, furthermore the surface is illuminated either by a light source emerging from the tip itself or an external lightning source. From the reflected light waves, NSOM determines the optical reflectivity of spatial structures. Through the tip’s scanning, a full map of the checked sample is obtained. The light captured by the tip is conducted to its backside to an optical fiber, connected to an optical detector, reading an electrical signal proportional to the captured light intensity in the tip. NSOM is also a technique for nanostructure to break the Near Field (NF) limit and get to high-resolution images, which are necessary for the nanostructure topics, as we use at the AFM. The motivation to develop the NSOM technique was followed by research by Ernst Abbe^31^, which developed a criterion for resolving two separate objects. Recently, combining the two separated methods – AFM and NSOM – was also presented^32,33^, as well as a proposal for triple-mode^34^.

In addition to these main two branches, several additional families of techniques exist. One of them, based on tip-enhanced optical effects, has emerged over the past years as a valuable alternative, which can successfully overcome the limitations of fluorescence-based super-resolution microscopy (f-SRM). Among the methods belonging to this family, one can find the scattering-type scanning the near-field optical microscopy (s-SNOM)^35^, the tip-enhanced Raman Spectroscopy (TERS)^36^, the tip-enhanced fluorescence (TEF)^37^, and the Second Harmonic Generation – Scanning Near-Field Optical Microscopy (SHG-SNOM). Being also part of this category, tip-enhanced photoluminescence (TEPL)^38^, or Photo-induced Force Microscopy (PIFM)^39^ have gained very high interest as they can extract optical properties at nanoscale resolution decided by the size of the tip used for scanning the sample for any wavelength of illumination. Moreover, they use very low-power excitation conditions and are not harmful to the investigated samples.

One can look after additional families, and identify the pros and cons in each one of them. The common part of all these techniques is the need for physical scanning. In this article, a new approach is presented for near-field surface imaging without the need to scan the sample. This new approach, entitled Near-field Projection Optical Microscope (NPOM), is presented for the first time. When compared to above existing techniques, this new approach is quite simple, using “projection” and not anymore “scanning”, in other words, “virtual” vs. “real” data acquisition. Presenting first the mathematical formalism, complementary series of numerical results are shown, while presenting an accurate reconstruction of the original object using patterns (sin, cos, and random). The accuracy of the reconstructed object will depend on two parameters: The number of iterations, and the usage (or not) of Moving-Average-Filter (MAF).

In this paper we show by both numerical simulation and proof of concept experiment the uniqueness of our technique.

## Working principle

Let P_n_(x) be a set of projected patterns (possibly even random ones) that form an orthogonal basis used for compressed sensing decomposition, with dimension N. We will denote by s(x) the shape of the object that is to be imaged.

If the readout value we get for each projected pattern of P_n_(x) is:

1 $$ r\left[ n \right] = \smallint s\left( x \right)P_{n} \left( x \right)dx $$

Then the reconstruction of the object from the set of readouts is given by:

2 $$ \hat{s}\left( x \right) = \mathop \sum \limits_{n} r\left[ n \right]P_{n}^{*} \left( x \right) $$

Let us prove the above claim. We start by substituting Eq. (1) into Eq. (2):

3 $$ \hat{s}\left( x \right) = \mathop \sum \limits_{n} \smallint s\left( {x^{\prime}} \right)P_{n} \left( {x{^{\prime}}} \right)P_{n}^{*} \left( x \right)dx{^{\prime}} = \smallint s\left( {x^{\prime}} \right)\left( {\mathop \sum \limits_{n} P_{n} \left( {x{^{\prime}}} \right)P_{n}^{*} \left( x \right)} \right)dx{^{\prime}} $$

By the orthonormality of the basis P_n_(x), one has:

4 $$ \mathop \sum \limits_{n} P_{n} \left( x \right)P_{n}^{*} \left( {x{^{\prime}}} \right) \approx \delta \left( {x - x{^{\prime}}} \right) $$

Substituting this relation into Eq. (3) yields the desired result:

5 $$ \hat{s}\left( x \right) = \smallint s\left( {x^{\prime}} \right)\delta \left( {x - x{^{\prime}}} \right)dx^{\prime} = s\left( x \right) $$

Let us now apply this theory to the specific case of the projections of grating:

6 $$ P_{n} \left( x \right) = exp\left( { - 2\pi in\nu_{0} x} \right) $$

Thus Eq. (4) becomes:

7 $$ \mathop \sum \limits_{n} exp\left( { - 2\pi in\nu_{0} x} \right)exp\left( {2\pi in\nu_{0} x{^{\prime}}} \right) = \mathop \sum \limits_{n} exp\left( { - 2\pi in\nu_{0} \left( {x - x^{\prime}} \right)} \right) $$

This is a Fourier series with coefficients a_n_ = 1. Thus Eq. (7) is equal to:

8 $$ \mathop \sum \limits_{n} exp\left( { - 2\pi in\nu_{0} \left( {x - x^{\prime}} \right)} \right) = \mathop \sum \limits_{n} \delta \left( {\left( {x - x^{\prime}} \right) - \frac{n}{{\nu_{0} }}} \right) $$

This means that the reconstructed signal will be periodic in space. Of course, our projected patterns are actually finite, so we will assume that the size of their supported domain is 1/$\nu_{0}$. These projected patterns can be expressed as:

9 $$ P_{n} \left( x \right) = rect\left( {x\nu_{0} } \right)exp\left( { - 2\pi in\nu_{0} x} \right) $$

Multiplying both sides of Eq. (8) by: $rect\left( {x\nu_{0} } \right)$ gives:

10 $$ rect\left( {x\nu_{0} } \right)\mathop \sum \limits_{n} exp\left( { - 2\pi in\nu_{0} \left( {x - x^{\prime}} \right)} \right) = \delta \left( {x - x^{\prime}} \right) $$

and therefore, we will obtain the desired reconstruction of Eq. (5) presented above.

In case that compressed sensing is to be applied, then the restoration problem with sparsity constraints is formalized as follows:

11 $$ \tilde{\theta }_{opt} = \mathop {\arg \min }\limits_{{\tilde{\theta }}} \mathop \sum \limits_{k = 1}^{K} y_{k} - A\tilde{\theta }_{2} + \tau \tilde{\theta }_{1} $$

where θ is the vector of coefficients representing the information in a given basis and y = Aθ is the captured data set, with A being the matrix relating the two. τis a weighting coefficient defined as part of the optimization criteria.

## Results

### Simulation results for 1-D object reconstruction algorithm

**Figure 1:**
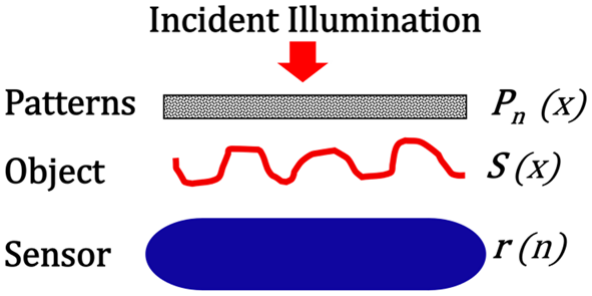
NPOM setup’s components.

As explained above, NPOM is a new method of near-field microscopy capable of super-resolution that eliminates the need for physical scanning. In its place, the method employs pattern projection and obtains a specific reading for each pattern (Eq. 1). The readings for each pattern and pattern function are then used to reconstruct the object (Eq. 2). It is possible to numerically demonstrate (in addition to the above analytical development) that the original object is well-reconstructed. If the method’s resolution is independent of the size of the sensor, it is dependent on the quality and the quantity of the patterns. Figure 1 presents a schematic of the setup.

As the patterns form an orthogonal array (Eq. 4), the higher the number of patterns we use, the more our approximation will approach the true object.

This is why, for the mathematical formalism visualization, we used intensity patterns and not phase patterns.

### Visualization conditions

In the above analytical development, a complex-valued harmonic pattern was used as the basis (Eq. 6). However, we must consider that actual sensors are only capable of providing information about the intensity, and not about the phase. If the pattern affects the phase alone, then the sensor will provide no information at all. For application purposes it is therefore preferable to use the following equation:

12 $$ r\left[ n \right] = \smallint s\left( x \right)P_{n} \left( x \right)dx = \smallint \left| {s\left( x \right)} \right|\left| {P_{n} \left( x \right)} \right|dx $$

The size of the pattern is finite, sharing a width of 1, while outside the pattern, the intensity is defined as 0. In this width, there are 10,000 pixels. The object is defined as a periodical and spatial function “rect”. All the functions are normalized, so the DC is 0 (it is always possible to subtract a fixed value in order to maintain this condition). The control of the visualization model is through 1) the number of patterns, 2) the type of patterns, and 3) the size of the object. The patterns used are *sin* and *cos* (we may need both to cover odd and even object functions) and also *random* patterns.

### Reconstruction quality criteria

As in any imaging process, we can expect that our reconstruction of the object will diverge somewhat from the original To limit this error, we will define two criteria used to quantify the reconstruction quality. The criteria will be based on the assumption that the objective function is square, with a height of $\pm 1$, so $\left| {s\left( x \right)} \right| = 1$. The criteria are defined as:

- **Modulation:** Reconstruction mean amplitude vs. the original one (Eq. 13):
  13 $$ m = \frac{{\left| {\hat{s}\left( x \right)} \right|}}{|s\left( x \right)} = \frac{{\left| {\hat{s}\left( x \right)} \right|}}{1} = \left| {\hat{s}\left( x \right)} \right| $$
- **RMS:** Reconstruction mean shape change after applying the correction of the amplitude size (Eq. 14):
  14 $$ RMS = \sqrt {\left( {\frac{{\hat{s}\left( x \right)}}{m} - s\left( x \right)} \right)^{2} } $$

### 1D visualization using sin and cos patterns

**Figure 2:**
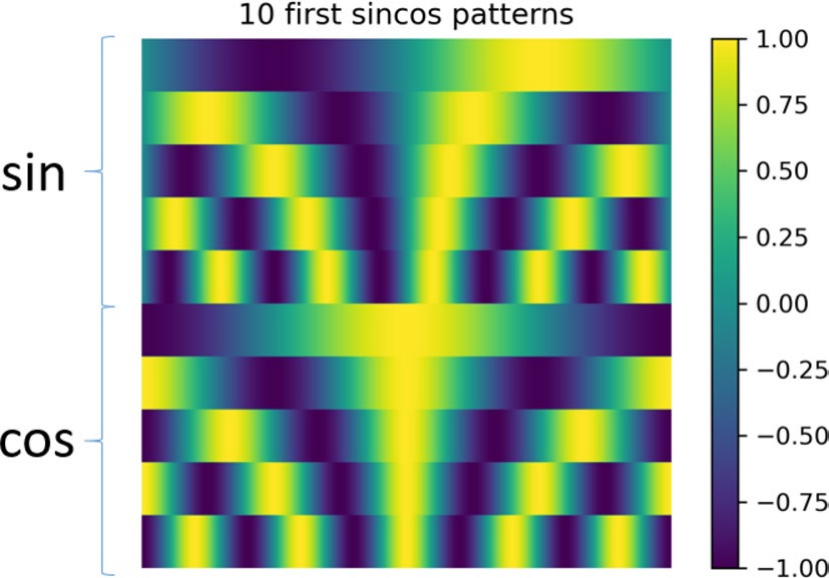
Visualization process: Images of the first ten sine and cosine patterns.

**Figure 3:**
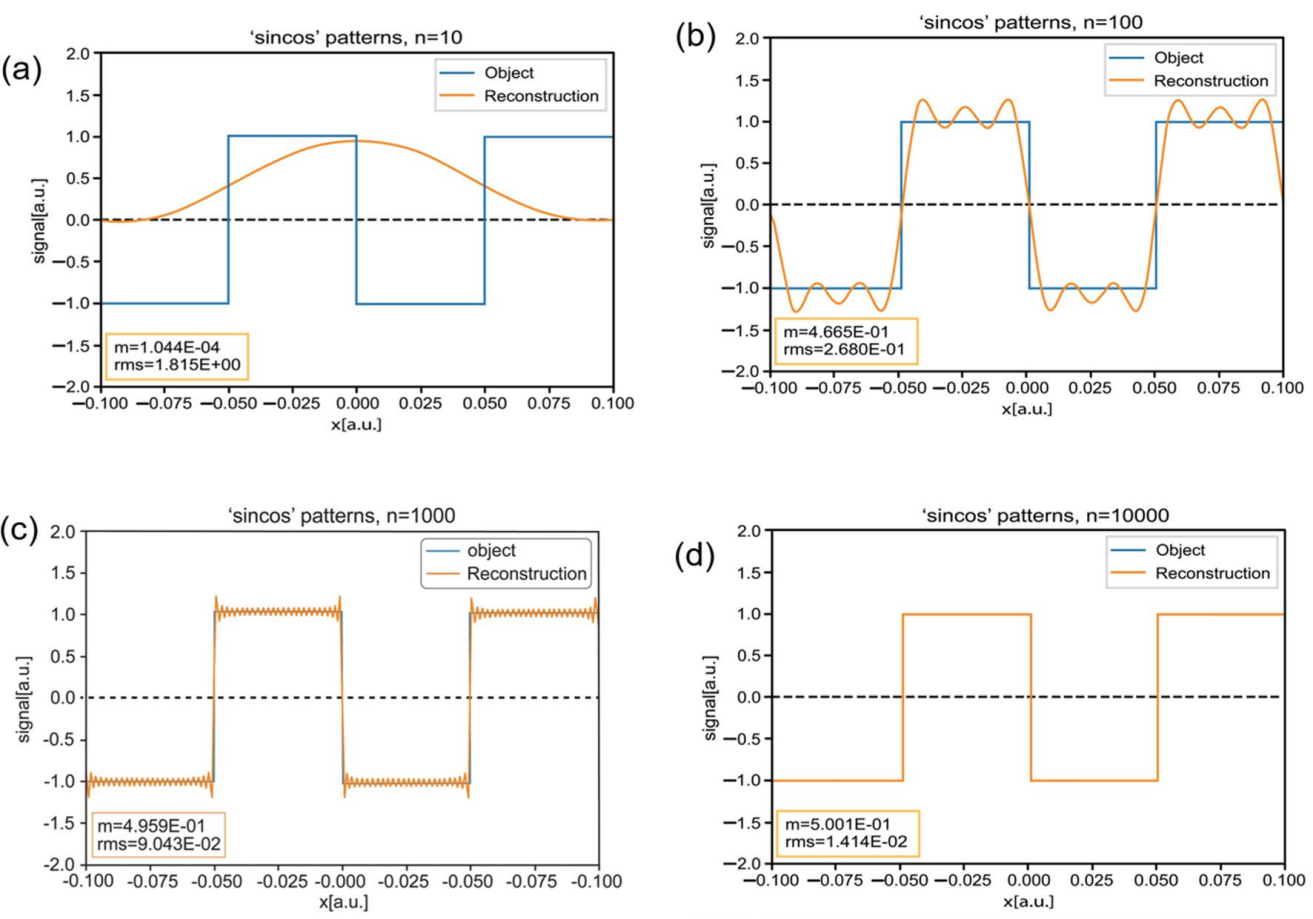
Reconstructed images for several representative numbers of patterns. (**a**) n = 10; (**b**) n = 100; (**c**) n = 1000; (**d**) n = 10,000.

To start, we consider a visualization process using sinusoidal patterns, as presented in Fig. 2. . The cosines support the reconstruction of even functions, while the sines form a basis for the odd ones. The first ten patterns – five sines and five cosines of varying frequency—are presented as images (Fig. 2). Reconstructed objects are presented in Fig. 3 for several representative numbers of patterns.

In theory, one can use any arbitrary amount of patterns to reconstruct any object of arbitrary width. For our purposes, we chose to work with an object whose width was set as one-tenth of the pattern size, and with numbers of patterns N that are multiples of 10. Of course, the reconstruction becomes more and more accurate as we increase the number of patterns used. The modulation increases while the RMS decreases.

### 1D Visualization using random patterns

**Figure 4:**
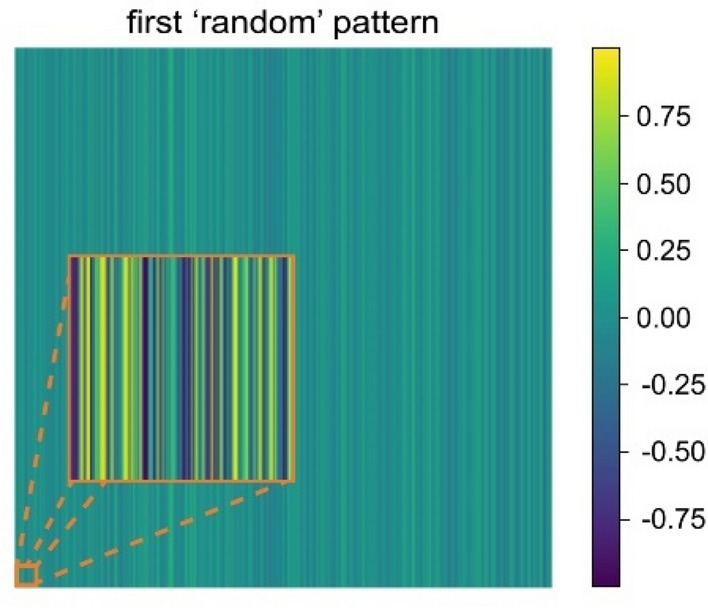
Visualization process: one of the random patterns presented as an image. As an insert, a zoom-in of the patterns is presented.

We now consider imaging using random patterns, an example of which is shown in Fig. 4.

**Figure 5:**
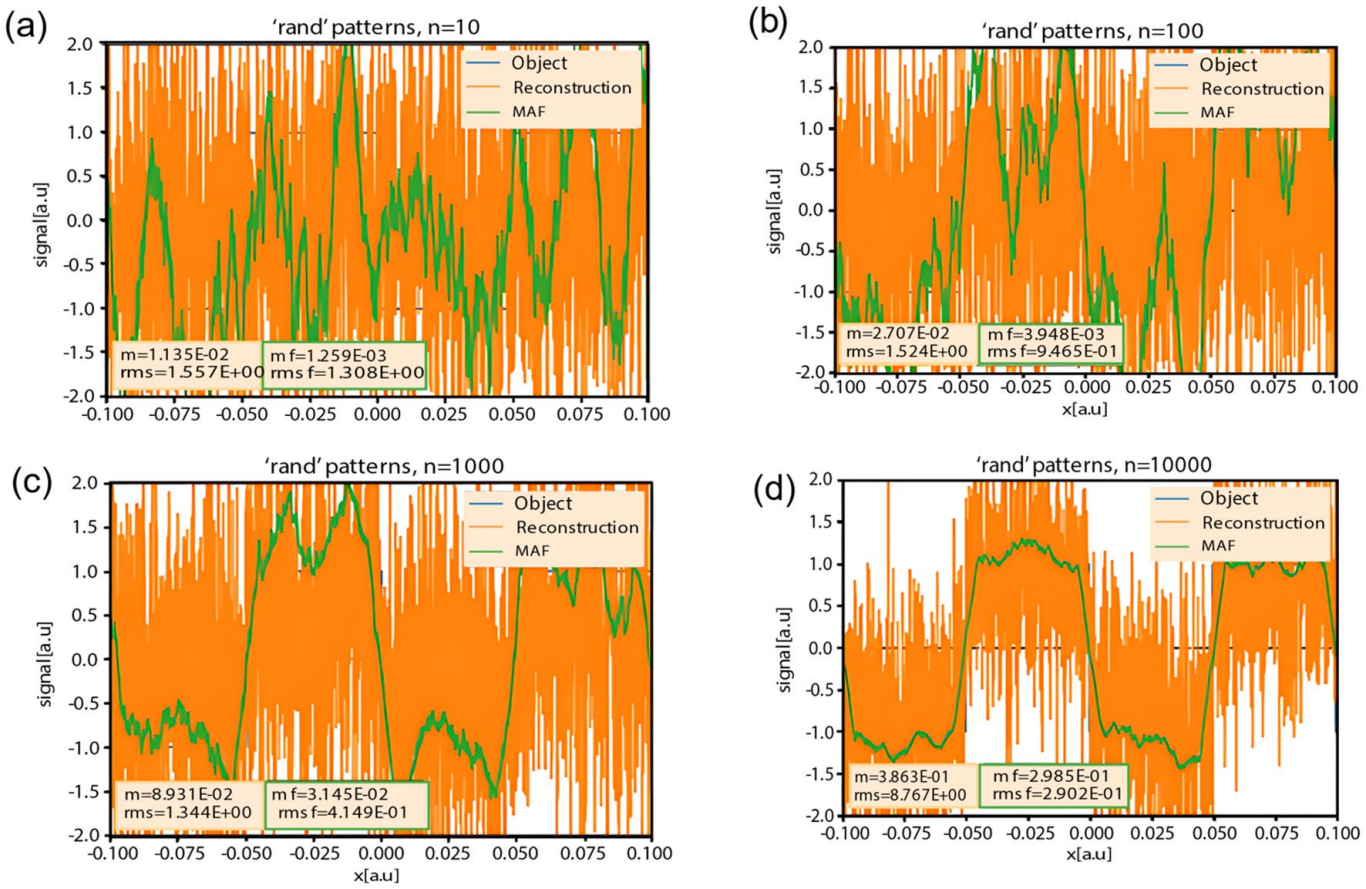
Reconstructed images for several representative numbers of patterns. (**a**) n = 10; (**b**) n = 100; (**c**) n = 1000; (**d**) n = 10,000.

As above, we chose to use an object with a width equal to one-tenth of the pattern size, and with a number of patterns that increase by multiples of ten. To reduce the randomness of the reconstruction, and to increase the accuracy, we employed a moving-average filter (MAF). One can observe that due to the MAF usage (green color), better results are obtained. However, it decreases high frequencies, so this affects the accuracy of the reconstruction. Again, the higher the number of patterns, the more accurate the reconstruction. Modulation increases and RMS decreases. Figure 5 shows the intensity profiles of the reconstructed images.

### Reconstruction quality vs. number of patterns

**Figure 6:**
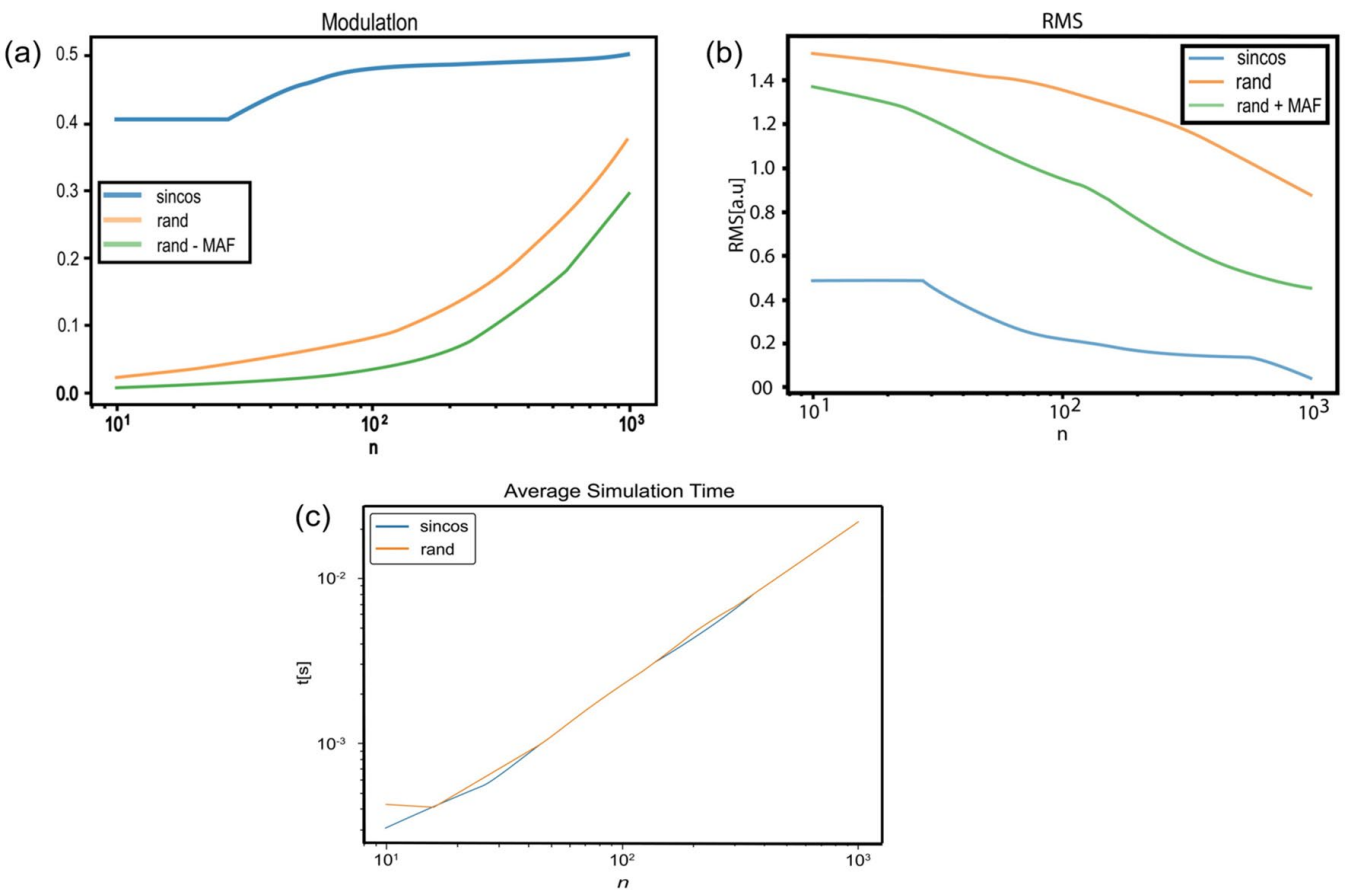
Reconstruction quality for different parameters. All graphs are expressed as a function of the number of iterations. (**a**) Modulation graph; (**b**) RMS graph. While the modulation is dimensionless since it expresses a ratio, the units of the RMS are the units of the signal [a.u.]; (**c**) Average simulation time. One can observe that the random patterns took longer at the beginning since it is the first run. Each curve represents an average of 100 simulation runs.

Figure 6 shows the effects that increasing the amount of sine, cosine, and random patterns has on the quality criteria defined above. One can observe that the sinusoidal patterns have good initial modulation and RMS values compared to the random ones and that both types of patterns gradually give more accurate results as we increase the number of them used. We also note that using MAF for the random patterns has a minimal effect on the modulation quality, while significantly increasing that of the RMS.

### Simulation results for two-dimensional (2D) object reconstruction

#### Increasing accuracy from 1 to 2D

**Figure 7:**
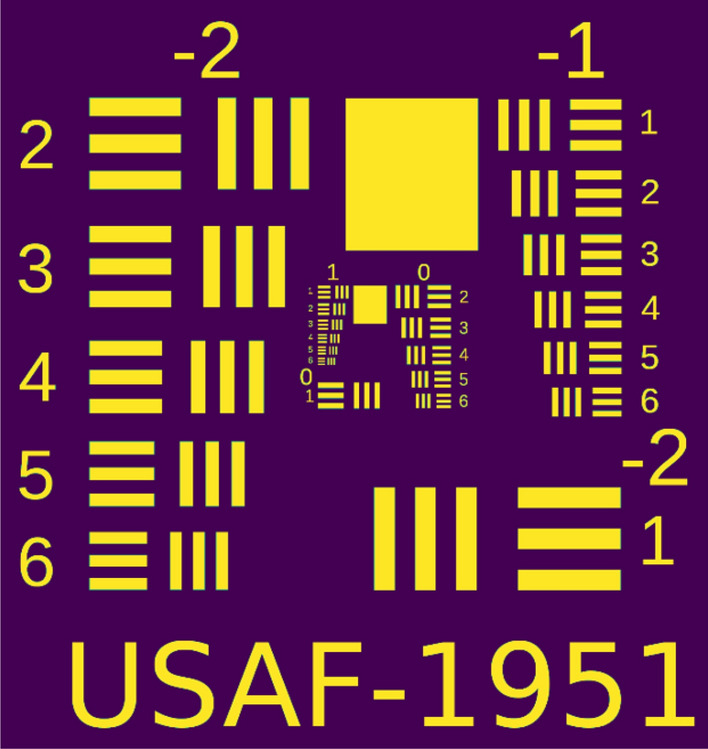
US Army Force resolution test chart.

It is trivial to extend the mathematical formalism developed above to the more realistic case of the reconstruction of a two-dimensional object. Of course, this time the reconstruction will require 2D patterns, which will increase the complexity of the visualization algorithm and decrease the rate of the reconstruction process. We chose the well-known USAF 1951 test chart, shown in Fig. 7, to serve as our two-dimensional sample. The object was originally defined by the U.S. Air Force MIL-STD-150A standard of 1951.

### 2D Visualization using sin and cos patterns

**Figure 8:**
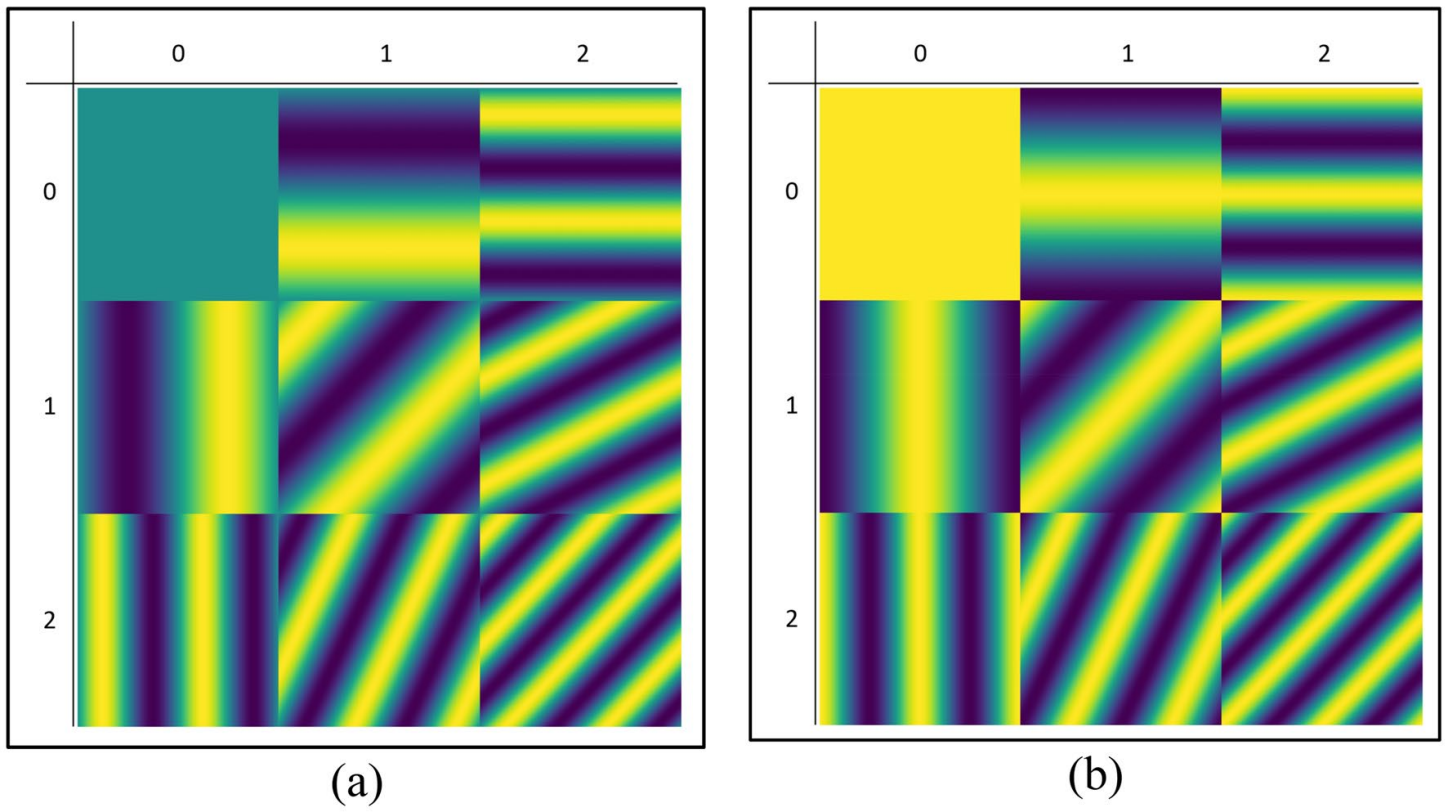
Separated *sin* and *cos* patterns. (**a**) Nine first *sin* patterns; (**b**) Nine first *cos* patterns.

As in the one-dimensional case, sines and cosines (Fig. 8) can also be employed in the reconstruction of a two-dimensional object. In the 2D case, the pattern has an independent frequency on each axis, and as a consequence, in order to obtain the same frequency result, one will need to use *n*^2^ patterns, instead of *n*. Since we need both sine and cosine patterns for each frequency, then the number of patterns will follow 2*n*^2^, where *n* is an integer. Moreover, when moving from 1 to 2D, the mathematical relations become:

15 $$ P_{n} \left( x \right) = \sin \left[ {2\pi \nu nx} \right] \to P_{nm} \left( {x,y} \right) = \sin \left[ {2\pi \left( {\nu_{x} nx + \nu_{y} my} \right)} \right] $$

16 $$ P_{n} \left( x \right) = \cos \left[ {2\pi \nu nx} \right] \to P_{nm} \left( {x,y} \right) = \cos \left[ {2\pi \left( {\nu_{x} nx + \nu_{y} my} \right)} \right] $$

In Fig. 8a the first nine (= 3^2^) sine patterns are presented, and in Fig. 8b the first nine (= 3^2^) cosine patterns patterns.

**Figure 9:**
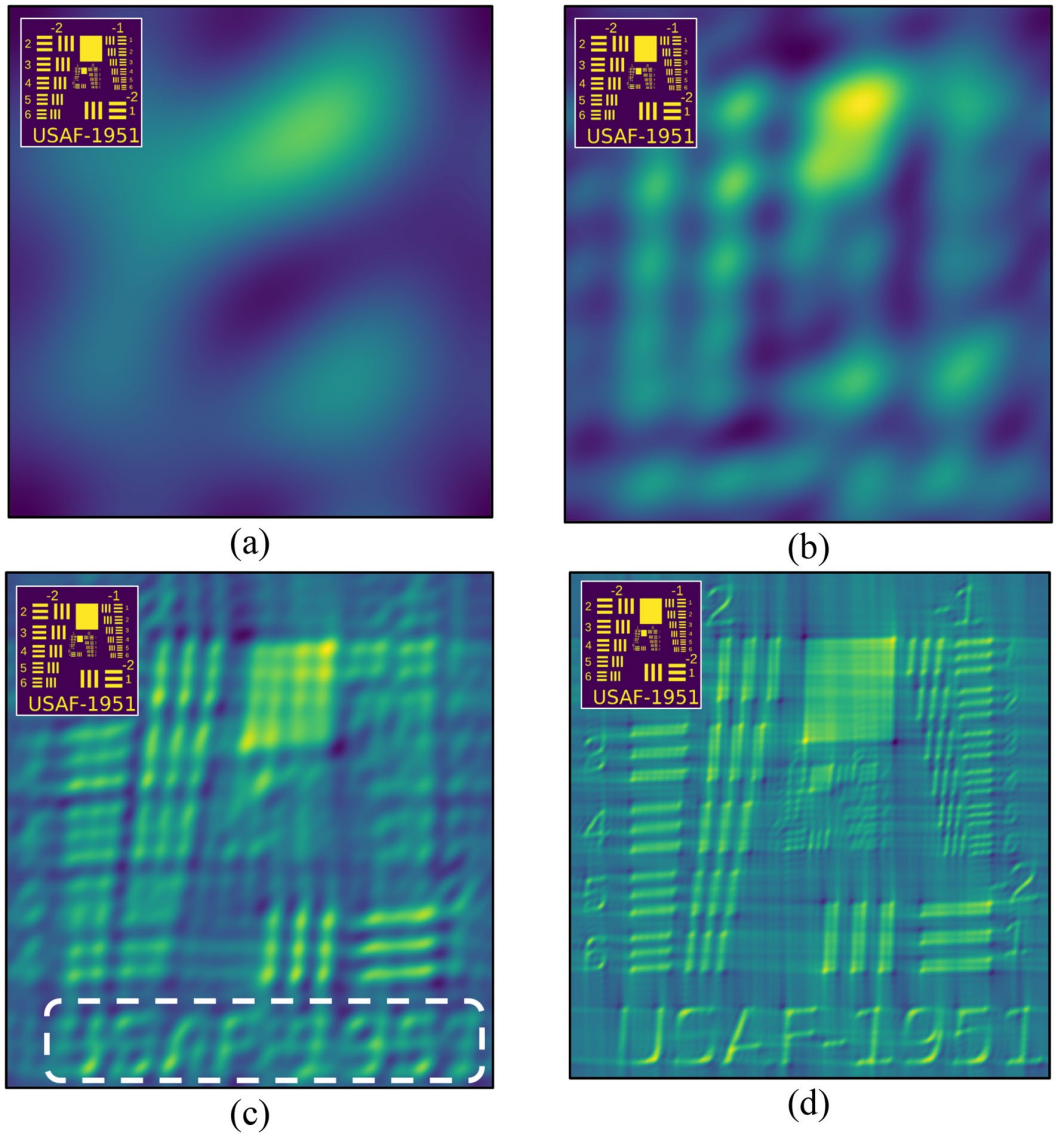
Combined *sin* and *cos* patterns. (**a**) n = 2 × 3^2^ = 18 ≈ order of of magnitude of 10; (**b**) n = 2 × 8^2^ = 128 ≈on the order of 100; (**c**) n = 2 × 23^2^ = 1058 ≈ on the order of of 1000; (**d**) n = 2 × 71^2^ = 10,082 ≈ on the order of 10,000.

The next step is to combine the sine and cosine patterns to obtain a much more accurate result, as presented in Fig. 9. Indeed, when compared to separate *sin* and *cos* patterns, one can discover the original shapes of the object (insert in Fig. 9), becomes more clear and accurate. The target patterns are not yet resolved in Fig. 9a ,b, but the “USAF-1951” caption has already emerged somewhat in Fig. 9c, and is readily legible in Fig. 9d. As predicted, the larger the number of iterations, the more accurate the results.

### 2D Visualization using random patterns

**Figure 10:**
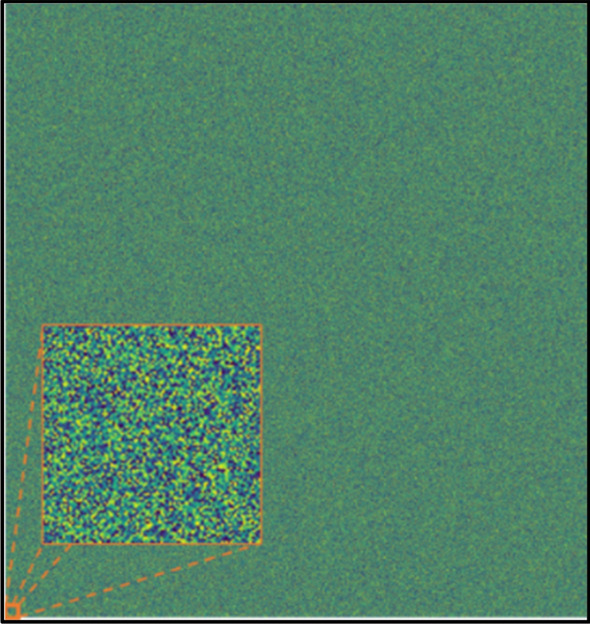
One of the *random* patterns used in the reconstruction series. As an insert, a zoom-in of the patterns is presented.

The logical next step is to ask whether we can do the same thing using 2D random patterns. The answer is positive, but this time, the process becomes more challenging, due to the randomness of the patterns (Fig. 10).

**Figure 11:**
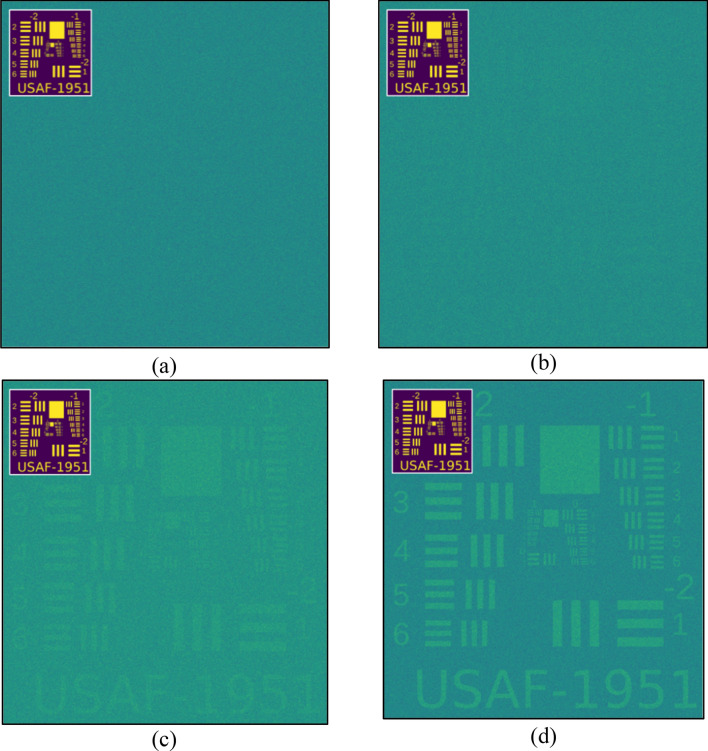
Usage of *random* patterns without Moving-Average-Filter (MAF). (**a**) n = 1000 ; (**b**) n = 10,000; (**c**) n = 100,000 ; (**d**) n = 1,000,000.

**Figure 12:**
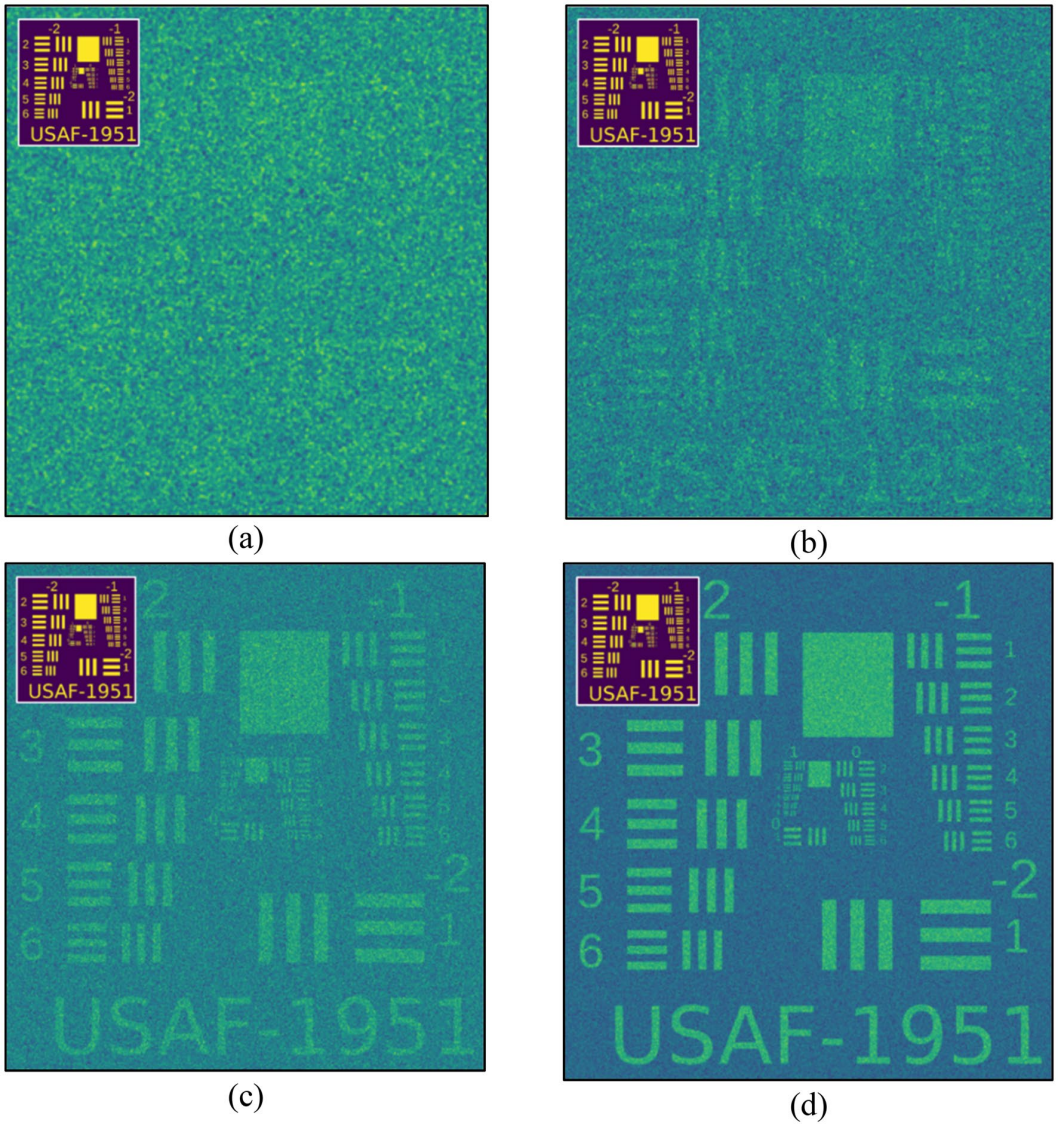
Usage of *random* patterns with Moving-Average-Filter (MAF). (**a**) n = 1000; (**b**) n = 10,000; (**c**) n = 100,000; (**d**) n = 1,000,000.

Two series of tests were performed: The first one used random patterns only, without the benefit of a moving average filter (Fig. 11), while the second series did employ a MAF. (Fig. 12). In the former case, we require a very large number of iterations before the pattern begins to emerge (Fig. 11), whereas, in the latter, the usage of a MAF enables a somewhate accurate reconstruction even with a relatively small amount (Fig. 12).

In Fig. 11a the target cannot be distinguished at all, and in Fig. 11b, one can barely notice the faint marks of the stripes, which only become somewhat noticeable in Fig. 11c. Without the use of a filter, it is necessary to increase the number of patterns up to several hundred thousand before the target becomes fully resolved as in Fig. 11d. In contrast, the use of a filter enables us to distinguish the target already at n = 1000 iterations (Fig. 12b), with even better quality than in Fig. 11b—a reduction of the number of patterns required for comparable resolution by ten times.

It can be seen that in order to obtain a good reconstruction with random patterns one must use a number of patterns several orders of magnitude greater than the number used for sine–cosine patterns. However, this is compensated for by the ease of generating random patterns as opposed to ones based on predetermined functions..

### Proof of concept experiment results

#### Experimental section

**Figure 13:**
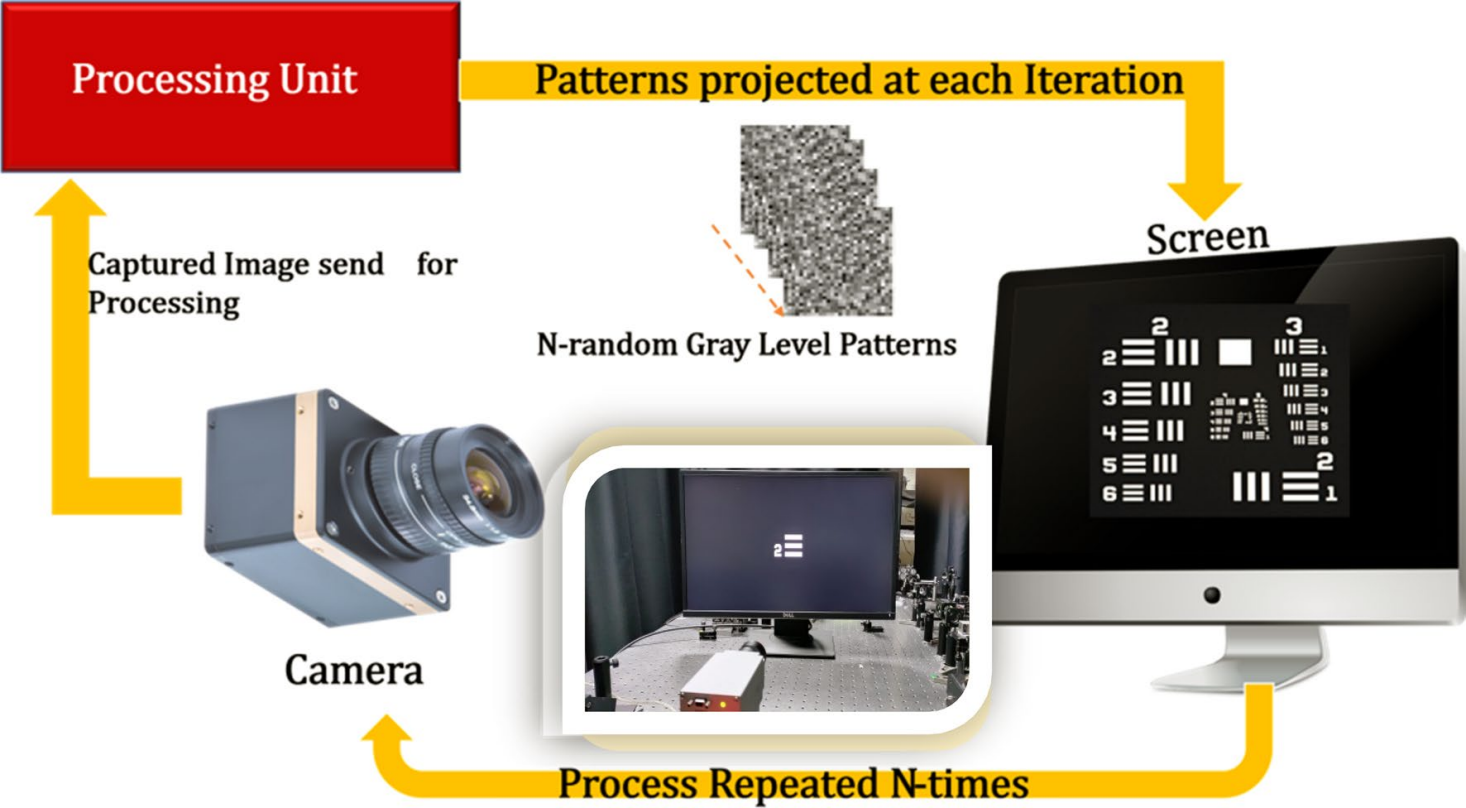
Schematic of the experimental setup. The inset shows the actual setup.

The proof of concept experiment was carried out with the aid of a computer screen as a means to project an object and eventually project this object with random gray-level patterns on top of it. The object was imaged using a camera (Pixelink, 6.5 µm pixel size) and lens as shown in schematic form in Fig. 13.

**Figure 14:**
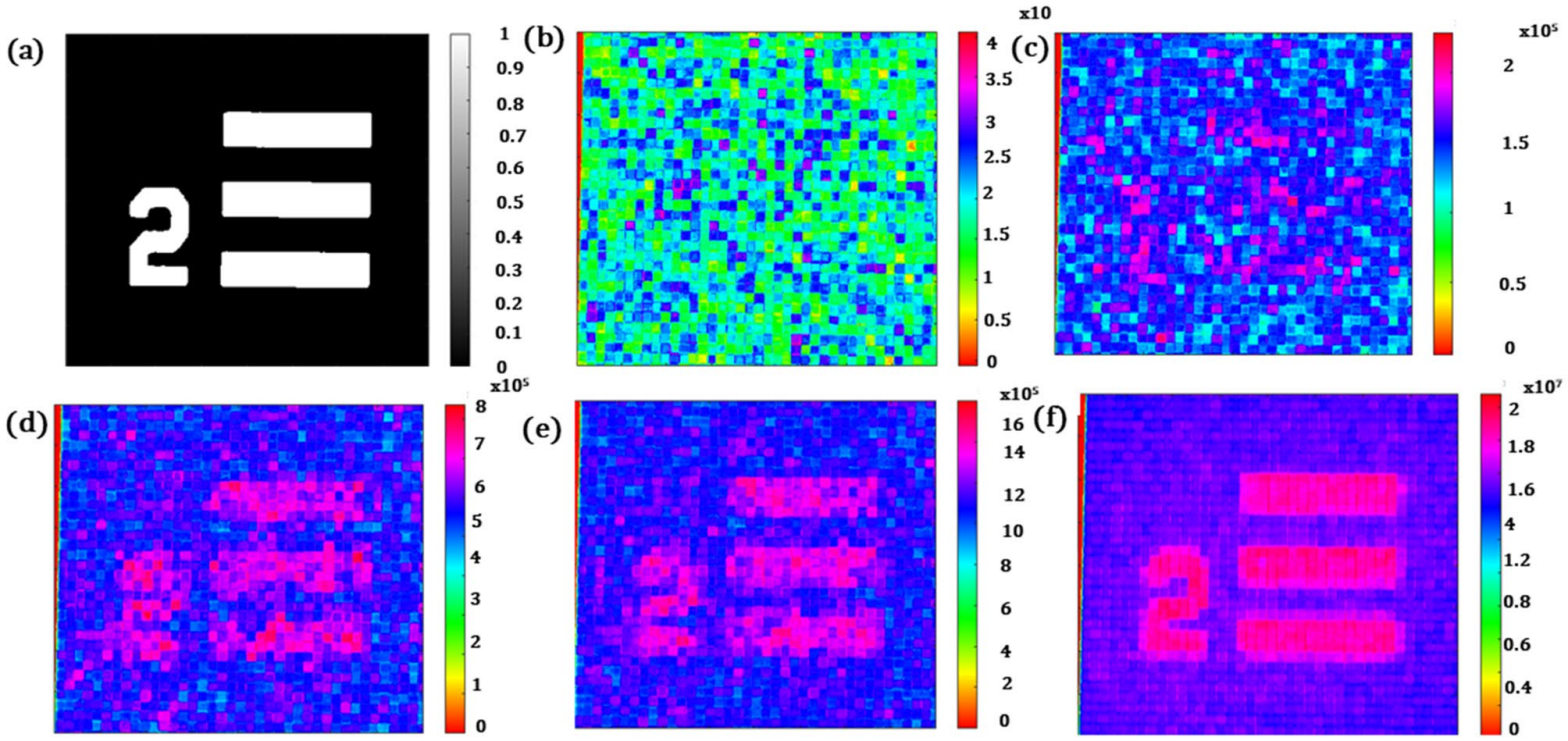
(**a**) High-Resolution Object, (**b**) Reconstruction after 100 iterations, (**c**) Reconstruction after 1000 iterations, (**d**) Reconstruction after 5000 iterations, (**e**) Reconstruction after 10,000 iterations, (**f**) Reconstruction after 150,000 iteration.

As in the case of the numerical simulations, a USAF-1951 target was used as an object, and several grayscale 400 × 400 random patterns were generated for use with it, However, we had effectively clubbed a certain number of pixels to create a binning of pixels. Although binning the pixels helps reduce the iteration number required for the reconstruction, when binning, it is necessary to confirm that the size of the clubbed pixel is enough to encode the structure of the object that we wish to reconstruct. In our case, we clubbed 10 × 10 pixels as one macro pixel, and hence the effective number of clubbed pixels was 40 × 40 out of 400 × 400 pixels. We projected around 150,000 patterns to get a perfect reconstruction. Please note that although we captured the image in a 2D sensor with a lens, we have used only a single mean average value of the total sensor intensity in the reconstruction process. In the reconstruction process, we have used a thresholding mechanism to convert all grayscale intensity patterns to binary values. The average intensity value of the original image was then subtracted from the average value of the image captured after the projection of a random pattern. This value was then multiplied with the projected pattern and eventually added up in each iteration, till we got a perfect reconstruction. Reconstructions corresponding to different numbers of iterations are shown in Fig. 14b–f.

It is clear from the experiments that the larger the number of projected patterns, the better the reconstruction. Figure 14a–f, shows the results obtained. Figure 14f is more resolved with a relatively good signal-to-noise ratio compared to Fig. 14e, with the number of projected patterns equaling 150,000 and 10,000 respectively.

**Figure 15:**
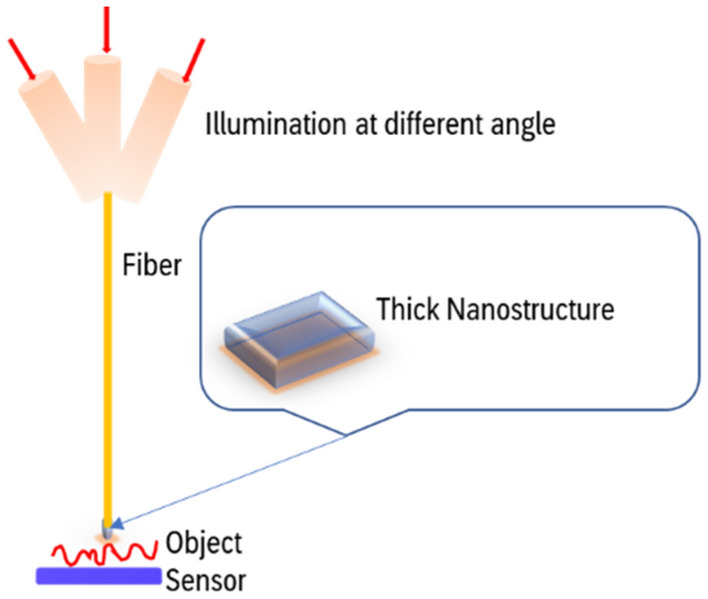
Schematic of the proposed implementation with a thick nanostructure illuminated at slightly different angles.

This proof of concept experiment can be directly applied to near-field microscopy. Indeed the final resolution is set by the resolution of the projected pattern. In the actual implementation of the idea, we would like to use a sufficiently thick nanostructure ( a volume diffraction grating) that can produce different patterns while illuminated at slightly different angles. There are several works of Peter B. Catrysse et al^40–42^, where they explored a new mechanism that allows efficient transport of light through subwavelength structures. Though the application is different the idea of a thick subwavelength structure was introduced earlier. The physical size of it would be in the order of the wavelength. This will be attached to the tip of the scanner. Since it is a volume structure the illumination at different angles would project different patterns on the object that is placed in close proximity to the nanostructure. The sensor is placed in proximity to the object (Fig. 15).

The reconstruction process would remain the same, and our detector would be a single pixel. It is worth noting that in our experiments we have used only the average value of all the CCD pixels to be in line with the idea of single-pixel detection.

## Conclusion

In this article, we presented a new method to analyze the near-field without the need for physical scanning. The method works via pattern projection and not by scanning using a nanometric probe. The main advantage of the proposed concept is that besides removing the need for a delicate mechanical scanning mechanism, the full field of regard/view is obtained simultaneously, and not point by point as in scanning-based methods. Furthermore, by using compressed sensing, the number of projected patterns that are needed to decompose the spatial information of the inspected object can be made smaller than the obtainable points of spatial resolution.

The mathematical formalism and a complementary numerical visualization were presented in order to showcase the power of the method. Our proof of concept experiment is a very close simulation of real implementation in near-field microscopy. We hope that our research can contribute to this innovative approach to non-scanning near-field imaging.

## Data Availability

The datasets used and/or analysed during the current study available from the corresponding author on reasonable request.

## References

[1] Zalevsky Z, Mendlovic D (2004). Optical Super Resolution.

[2] Z. Zalevsky, D. Mendlovic, A. W. Lohmann, Progress in optics, Vol. XL, Ch. 4: Optical system with improved resolving power. (1999).

[3] Mendlovic D, Lohmann AW, Konforti N, Kiryuschev I, Zalevsky Z (1997). One dimensional superresolution optical system for temporally restricted objects. Appl. Opt..

[4] Shemer A, Zalevsky Z, Mendlovic D, Konforti N, Marom E (2002). Time multiplexing super resolution based on interference grating projection. Appl. Opt..

[5] Garcia J, Zalevsky Z, Fixler D (2005). Synthetic aperture superresolution by speckle pattern projection. Opt. Exp..

[6] Zalevsky Z, Saat E, Orbach S, Mico V, Garcia J (2008). Exceeding the resolving imaging power using environmental conditions. Appl. Opt..

[7] Zalevsky Z, Fish E, Shachar N, Vexberg Y, Micó V, Garcia J (2009). Super resolved imaging with randomly distributed, time and size varied particles. JOPA A.

[8] Gur A, Fixler D, Micó V, Garcia J, Zalevsky Z (2010). Linear optics based nanoscopy. Opt. Exp..

[9] Gur A, Zalevsky Z, Micó V, García J, Fixler D (2011). The limitations of nonlinear fluorescence effect in super resolution saturated structured illumination microscopy system. J. Fluoresc..

[10] Zalevsky Z, Zlotnik A (2008). Axially and transversally super resolved imaging and ranging with random aperture coding. JOPA A.

[11] Binnig G, Quate CF, Gerber C (1986). Atomic force microscope. Phys. Rev. Lett..

[12] Binnig G, Rohrer H (1986). Scanning tunneling microscopy. IBM J. Res. Dev..

[13] Binnig G, Rohrer H (1987). Scanning tunneling microscopy - from birth to adolescence. Rev. Mod. Phys..

[14] Salapaka S, Salapaka M (2008). Scanning probe microscopy. IEEE Cont. Syst. Mag..

[15] Bowen J, Cheneler D (2017). Selecting suitable image dimensions for scanning probe microscopy. Surfaces Interfaces.

[16] C. M. Harris, The saga of AFM, a journey into a hot analytical market. *Anal. Chem.*, 627–635 (2001). 10.1021/ac012631x 11721950

[17] Betzig E, Lewis A, Harootunian A, Isaacson M, Kratschmer E (1986). Near-field scanning optical microscopy (NSOM), development and biophysical applications. Biophy. J..

[18] C. M. Harris, Shedding light on NSOM. Anal. Chem. 223–228 (2003). 10.1021/ac0313264 12751532

[19] Ozcan A, Cubukcu E, Bilenca A, Crozier KB, Bouma BE, Capasso F, Tearney GJ (2006). Differential near-field scanning optical microscopy. Nano Lett..

[20] Jiang R-H, Chen C, Lin D-Z, Chou H-C, Chu J-Y, Yen T-J (2018). Near-field plasmonic probe with super resolution and high throughput and signal-to-noise ratio. Nano Lett..

[21] Chouhan S, Bajpai AK, Bhatt R (2019). Analysis of topographical parameters and interfacial interaction of zinc oxide reinforced poly (vinyl alcohol-g-acrylonitrile) nanocomposite film surfaces using atomic force microscopy. Nano-Struct. Nano-Obj..

[22] Oladapo BI, Malachi IO, Malachi OB, Elemure IE, Olawumi AM (2020). Nano-structures of 4D morphology surface analysis of C1.7Mn0.6P0.1S0.07 (SAE 1045) tool wear. Nano-Struct. Nano-Obj..

[23] Hutter JL, Bechhoefer J (1994). Measurement and manipulation of van der Waals forces in atomic-force microscopy. J. Vac. Sci. Technol. B.

[24] Jang J, Schatz GC, Ratner MA (2004). Capillary force in atomic force microscopy. J. Chem. Phys..

[25] Kohoutek J, Wan IYL, Mohseni H (2010). Dynamic measurement and modeling of the Casimir force at the nanometer scale. Appl. Phys. Lett..

[26] Malotky DL, Chaudhury MK (2001). Investigation of capillary forces using atomic force microscopy. Langmuir.

[27] Belaidi S, Girard P, Leveque G (1997). Electrostatic forces acting on the tip in atomic force microscopy: Modelization and comparison with analytic expressions. J. Appl. Phys..

[28] Hosaka S, Shintani T, Kikukawa A, Itoh K (1999). Nano-optical image and probe in a scanning near-field optical microscope. Appl. Surf. Sci..

[29] Karelits M, Mandelbaum Y, Chelly A, Karsenty A (2017). Electro-optical study of nanoscale Al-Si truncated conical photodetector with subwavelength aperture. J. of Nanoph..

[30] Karelits M, Mandelbaum Y, Chelly A, Karsenty A (2018). Laser beam scanning using near-field scanning optical microscopy nanoscale silicon-based photodetector. J. Nanoph..

[31] Abbe E (1881). On the estimation of aperture in the microscope. J. R. Microsc. Soc..

[32] Karelits M, Lozitsky E, Chelly A, Zalevsky Z, Karsenty A (2019). Advanced surface probing using dual-mode NSOM-AFM silicon-based photo-sensor. Nanomaterials.

[33] Z. Zalevsky, A. Karsenty, A. Chelly, Photodetector for scanning probe microscope, Patent No. US 11,169,176 B2. Date of Patent: Nov. 9, 2021.

[34] Karelits M, Zalevsky Z, Karsenty A (2020). Nano polarimetry: Enhanced AFM-NSOM triple-mode polarimeter tip. Nat. Sci. Rep..

[35] X. Chen, Y. Zhang, L. Wang, Y. Wang, X. Zhang, Modern scattering-type scanning near-field optical microscopy for advanced material research, *Adv. Mater*. 1804774 (2019). 10.1002/adma.201804774 30932221

[36] Verma P (2017). Tip-enhanced Raman spectroscopy: Technique and recent advances. Chem. Rev..

[37] Gerton JM, Wade LA, Lessard GA, Ma Z, Quake SR (2004). Tip-enhanced fluorescence microscopy at 10-nanometer resolution. Phys. Rev. Lett..

[38] Yang B (2020). Sub-nanometre resolution in single-molecule photoluminescence imaging. Nat. Photon..

[39] Nowak D (2016). Nanoscale chemical imaging by photoinduced force microscopy. Sci. Adv..

[40] Catrysse PB, Fan S (2008). Propagating plasmonic modes in nano-scale apertures and its implications for extraordinary transmission. J. Nanophoton..

[41] P. B. Catrysse, H. Shin, and S. Fan, "Propagating modes in subwavelength cylindrical holes," J. Vac. Sci. Technol. B 23, 2675, 2005. [pdf]

[42] Shin H, Catrysse PB, Fan S (2005). The effect of propagating modes on the transmission properties of subwavelength cylindrical holes. Phys. Rev. B.

